# Association of HLA‐DQ4/5 genotype polymorphisms with celiac disease in a group of children in Southwest Iran: A case‐control study

**DOI:** 10.1002/hsr2.2242

**Published:** 2024-07-14

**Authors:** Ali Keshtkari, Marzieh Danaei, Milad Mollaali

**Affiliations:** ^1^ Department of Pediatrics, School of Medicine and Clinical Research Center, Emam Sajad Hospital Yasuj University of Medical Sciences Yasuj Iran; ^2^ Member of Iran High‐Tech Laboratory Network Dana Gene Pajoohan Karmania Company Kerman Iran; ^3^ Department of Biology, Faculty of Science University of Sistan and Baluchestan Zahedan Iran

**Keywords:** celiac disease, genetic variation, HLA‐DQ alpha‐Chains, HLA‐DQ beta‐chains, polymerase chain reaction

## Abstract

**Background and Aim:**

Celiac disease (CD) has proinflammatory and pathogenic immune responses to gluten in intestinal tissue, leading to structural changes in the mucosa of the small intestine. The association of human leukocyte antigen (HLA)‐DQ2 and DQ8 genotypes with CD has been previously reported. This test has a negative predictive value close to 100%, so its main purpose is to rule out the detection of CD completely or almost completely. There is limited information regarding HLA‐DQ4/5 in CD. This study was conducted to determine the HLA‐DQ4/5 genotypes in a group of Southwestern Iranian children with CD.

**Methods:**

We conducted a case‐control study in Southwest Iran involving 100 participants, employing a nonprobabilistic sampling method. Samples were taken from participants' oral buccal mucosa at Imam Sajjad Hospital of Yasuj, Iran. Then DNA was extracted from these samples and used to determine the frequency of HLA‐DQ4/5 genotypes through Sequence‐Specific Primer‐Polymerase Chain Reaction assay. SPSS 20 was utilized for statistical analyses.

**Results:**

Fifty diagnosed patients with CD (high anti‐tissue transglutaminase [tTG]‐IgA level [upper limit of normal] with pathological findings of Marsh III) and 50 non‐CD individuals (normal anti‐tTG‐IgA level and normal total IgA level) were enrolled in the study from August 5, 2022 to October 15, 2023. Findings showed that the DQ4a*4b allele has the highest frequency in the CD samples (78%, *p* < 0.01) followed by the DQ5a*5b allele (12%, *p* < 0.01). Additionally, there was a higher prevalence of DQ4/DQ5 in patients with CD compared to controls (odds ratio = 6.5, confidence interval = 0.84 to 69.46, *p* < 0.01). Furthermore, a significant association was found among HLA DQ4/5 genotype, age (>9.5) (*p* < 0.01), and gender (female) (*p* < 0.05).

**Conclusion:**

The observed significant differences among HLA‐DQ4 and HLA‐DQ5 in Iranian CD samples against controls and the high value of the relative risks showed the significant function of the studied alleles in the prevalence of CD in Iranian patients.

## 1 INTRODUCTION

Celiac disease (CD) is a complex disorder influenced by various genetic and environmental elements that determine its manifestation. The exact mechanisms behind CD remain unclear; however, there is an indication that it involves an autoimmune response triggered by gluten.^1^ Gluten, which is a major protein component of barley, rye, and wheat in the diet, can alter the normal behaviors of T‐cell receptors in the duodenum mucosa, which is a primary factor in triggering autoimmune responses in CD patients.^2^ Consequently, CD results in inflammation of the small intestinal mucosa, and the enhanced atrophy of the villus could prevent nutrient uptake.^3^ CD can also be determined via a combination of specified (human leukocyte antigen [HLA] and non‐HLA) genes, infectious diseases in the first years of life, and modifications in the gut microbiome.^4^, ^5^ Disruptions in at least 42 non‐HLA genes, such as STAT4, CTLA4, and IL2, have been reported to contribute to CD progress.^6^

The latest update to the guideline from the European Society for Pediatric Gastroenterology, Hepatology, and Nutrition (ESPGHAN) in 2020, outlines the criteria for diagnosing CD with or without a biopsy of the duodenum. According to this guideline, if the transglutaminase 2 (TGA)‐IgA level (which is equivalent to tissue transglutaminase [tTG]‐IgA) exceeds 10 times the upper limit of normal (≥10 × ULN) and also the endomysial antibodies (EMA)‐IgA test is positive, a no‐biopsy approach is recommended. On the other hand, if the TGA‐IgA level is less than 10 times the upper limit of normal (<10 × ULN), a biopsy is necessary to minimize the risk of a false‐positive diagnosis.^7^, ^8^ An analysis of children with CD found that the presence of Hashimoto thyroiditis, Turner syndrome, Down syndrome, diabetes mellitus, and hypothyroidism all contributed to the possibility of CD in these children.^9^ To mitigate the risk of enduring complications, such as malignancy, stunted growth, infertility, and reduced bone density, it is imperative to diagnose CD as early as possible and initiate treatment.^10^, ^11^ Single‐nucleotide polymorphism (SNP)‐based assays are economical and effective at detecting common variants associated with CD, such as HLA‐DQ7.5, DQ2.2, DQ8, and DQ2.5. However, to detect rare variant alleles, detailed allele typing is necessary. Consequently, the precision in ruling out CD through HLA testing is still contingent on the technique employed.^7^ A wide range of samples with CD having the class II of the HLA genes including HLA‐DQ2.5 (DQA1*05‐DQB1*02) and HLA‐DQ8 (DQA1*03‐DQB1*0302).^12^ A great relationship was reported between HLA‐DQ2.5 and CD prevalence, associated with its dependency on attaching gluten proteins.^13^ However, HLA‐DQ2.2 showed a weak association with CD prevalence.^14^ Furthermore, the CD frequency is determined to be higher in homozygous samples for the HLA‐DQ2.5 or HLA‐DQ2.5/DQ2.2 genotypes in comparison with homozygous samples for HLA‐DQ2.2 or heterozygous for HLA‐DQ2.5 or DQ2.2.^15^, ^16^ In European nations, the combined allele frequency of HLA‐DQ2 (90%) and DQ8 (5−10%) are almost 100%.^17^

The prevalence of CD among the general population varies from 0.5% to 2%, averaging around 1%,^18^ yet this 1% rate nearly doubles when screening projects are carried out among school‐aged children.^19^ The ratio of affected females to males in CD varies from 1:3 to 1.5:1; this disease is known to affect individuals of all ages, with more than 70% of new diagnoses occurring in individuals over 20 years old.^9^, ^20^ European nations (such as Finland and Sweden) and people of European ancestry demonstrate an increased susceptibility to developing CD in contrast to other geographic areas.^21^ Additionally, The prevalence of CD is different among countries, and it is believed to affect approximately 1% of the overall population in Iran.^22^

Based on our information no study has been performed about the link of HLA‐DQ4/5 genotypes and CD in Iran. Therefore, for the first time, the frequency of the CD‐associated genes including HLA‐DQ4 and DQ5 in 50 children with CD compared to 50 control individuals was evaluated.

## 2 MATERIALS AND METHODS

### 2.1 Sample collection

The current case‐control study was conducted in the Western Iranian population using a nonprobabilistic sample selection strategy. The study population contained 100 participants; the case group was 50 CD patients who had an anti‐tTG‐IgA positive test (upper limit of normal), and their pathological findings of the small intestine's mucosal damage were Marsh III. Furthermore, the control group was 50 non‐CD individuals without any CD history, cancer, or autoimmune disorders, and also their anti‐tTG‐IgA and total IgA levels' test results were in the normal range. CD patients who were >15 years old and Marsh I or Marsh II were excluded. Swab samples were taken from the oral buccal cells of each participant at Emam Sajad Hospital of Yasuj, Iran. Samples were kept in a stabilizing buffer for further analysis. All the experiments were confirmed by the Ethical Commission of Yasuj University of Medical Sciences (Approval ID: IR.YUMS.REC.1402.113); also, written informed consent was obtained from all participants.

### 2.2 DNA extraction

The DNA extraction procedure was done as reported before.^23^ Briefly, the oral buccal cell samples were solved in 0.5 mL of the extraction buffer (containing 10 mM Tris with pH 8.0, EDTA [10 mM], and 2.0% SDS), and 50 μL of 10% SDS, besides 5–10 μL 20 mg/mL of proteinase K (Sinaclon). The samples were kept for 1–2 h at 58°C. After that, 0.5 mL of phenol:chloroform:isoamyl alcohol solution with a ratio of 25:24:1 was added and inverted for 3 min. Then, centrifugation of the samples (Sigma) for 10 min with 10,000*g* (4°C), and the top layer of the mixture was moved to another tube. This step was repeated twice and following this, the double volume of the removed liquid cold isopropanol (Merck) was mixed, besides 1/10 volume of sodium acetate (3 M), and kept at freezer (−20°C) for 2 h. Afterward, the samples were centrifuged at 9000*g* (4°C) for 8 min, and following the supernatant decanting, 250 μL of 70% ethanol (Hamoon Teb) was mixed, and the pellet was dissolved. In this regard, the centrifugation of the mixture was done at 9000 rpm for 8 min. Finally, the pellet was mixed with 30 μL of DNase‐free H_2_O and kept in the freezer (−20°C) or for the following procedure. The accuracy and quantity of the isolated DNA were assessed by 1% agarose gel and spectrophotometry (260/280 nm absorbance).^24^, ^25^, ^26^

### 2.3 Genotyping

To genotype‐specific HLA‐DQ4/5 alleles, specific primers were designed for the 4a/4b and 5a/5b locus using published primers.^27^ The accuracy and specificity of the primers were checked by the BLAST database in NCBI (https://blast.ncbi.nlm.nih.gov/Blast.cgi). The primer sequences are presented in Table 1. Designed primers were synthesized by Sinaclon Company (Tehran, Iran). After that, DNA samples were used to amplify the desired sequences by Sequence‐Specific Primer‐Polymerase Chain Reaction assay based on the program as follows 94°C for 4 min, 10 cycles at 94°C for 25 s, 59°C for 25 s, 72°C for 30 s, and 25 cycles at 94°C for 25 s, 60°C for 25 s and 72°C for 30 s. The resulting products were evaluated by a 1% agarose gel electrophoresis.

**Table 1:** Sequences of primers used for HLA typing.

Primer name	Sequence 5’ → 3'	Product length (bp)
4a	CTACTTCACCAACGGGACC	204
4b	GTAGTTGTGTCTGCATACGG	
5a	GACGGAGCGCGTGCGGGG	218
5b	GCTGCAGGATCCCGCGGT	

Abbreviations: bp, base pair; HLA, human leukocyte antigen.

### 2.4 Data analysis

The acquired findings were analyzed using the SPSS software (Version 20). The Chi‐Square Test was used to compare genotypes in age and gender. Statistical significance was determined at the *p* ≤ 0.05.

## 3 RESULTS

In this study, 50 CD patients (35 men and 15 women with an average age of 9.5 years, range 1–15) and 50 normal individuals (30 men and 20 women with an average age of 25 years, range 18–27) with Iranian ethic were assessed regarding HLA‐DQ4 and DQ5 genes between August 5, 2022 and October 15, 2023. The frequencies of allele and genotype of DQ4 and DQ5 genes in patients were evaluated against the normal individuals. The allele frequency of the DQ4a*4b was significantly elevated in CD patients against the non‐CD individuals (78% against 22%; *p* < 0.01). The frequency of the DQ5a*5b allele was also increased in patients against controls (12% against 4%; *p* < 0.01) (Table 2).

**Table 2:** HLA‐DQ4 (4a*4b) and HLA‐DQ5 (5a*5b) allele frequencies in patient and control groups.

Allele	Patients (*n* = 50) (100 alleles)		Controls (*n* = 50) (100 alleles)		
	*n*	Frequency (%)	*n*	Frequency (%)	*p* Value
4a*4b	78	78	22	22	<0.01**
5a*5b	12	12	4	4	<0.01**

Abbreviation: HLA, human leukocyte antigen.

** Significant at *p* < 0.01.

Evaluation of DQ4 and DQ5 genes revealed that 23 of 50 CD patients had DQ4 in homozygous status with 46% frequency, while this genotype was observed in five normal individuals (10%). The prevalence of the DQ4/DQ5 was elevated in patients in comparison with controls (10% vs. 2.1%) (Table 3).

**Table 3:** Frequency of HLA‐DQ4/DQ5 genotype in patient and control groups.

HLA genotype	Patients		Controls		Odd ratio	95% CI	Relative risk	*p* Value
	*n*	Frequency (%)	*n*	Frequency (%)				
DQ4/DQ5	23	46	5	10	6.5	0.84‐69.46	1/6	<0.01**

Abbreviation: HLA, human leukocyte antigen.

** Significant at *p* < 0.01.

In the next phase of our study, the association of DQ4/DQ5 genotypes with the age and frequency of patients was analyzed. Based on our results, the frequency of the patients carrying DQ4 and DQ5 alleles allele was higher in those who were >9.5 years old. Moreover, there was an association with the high frequency of female patients with DQ4/DQ5 alleles (Table 4).

**Table 4:** Frequency of HLA‐DQ4 and DQ5 alleles based on patients' age and gender.

Characteristics		DQ4		DQ5		
		*n*	Frequency (%)	*n*	Frequency (%)	*p* Value
Age	>9.5	5	5	2	2	<0.01**
	<9.5	45	45	48	48	
Gender	Male	22	22	10	10	<0.05*
	Female	28	28	40	40	

Abbreviation: HLA, human leukocyte antigen.

* Significant at *p* < 0.05.

** Significant at *p* < 0.01.

## 4 DISCUSSION

CD is a systemic immune‐associated disease resulting from gluten‐containing grains intolerance in persons who are susceptible genetically.^28^ It has been reported that there is a great association between wheat utilization and the frequency of HLA‐DQ2 and DQ8 over the world.^29^ The CD history is related to the wheat cultivation spreading following the revolution of agriculture. The CD prevalence is associated with the HLA class II related genes: HLA‐DQ2 (DQA1*05‐DQB1*02) and HLA‐DQ8 (HLA‐DQA1*0301‐DQB1*0302) as the gluten specified genes.^30^ On the contrary, the lack of HLA‐DQ2 or HLA‐DQ8 showed a 100% negative expectation ratio in excluding the detection of CD.^4^

The present research showed that in 50 Iranian children patients referred to the Emam Sajad Medical Center, there were 78% had the DQ4a*04b allele and 22% had the DQ5a*5b allele. Moreover, the frequency of the HLA DQ4/DQ5 genotype was higher in CD patients. These results are similar to the findings of studies about CD samples from different Arab areas about HLA‐DQ2 variants as follows: in Egypt (76.43%), Gaza Strip (85.7%), Jordan (100%), and Morocco CD patients (44.3%).^31^, ^32^, ^33^, ^34^ Furthermore, the HLA‐DQ2 variants showed high frequency in CD patients of French (87%), Italian (84%), and England (88%).^35^ A study conducted among pediatric CD patients in Albania revealed that the frequency of HLA‐DQ2/DQ8 haplotype was 95.0%.^36^ Sahin and Mermer's study reported the frequency of HLA‐DQ genotypes (95%) in CD within a Turkish population; they found that 57.9% of patients had HLA‐DQ2, while HLA‐DQ2/DQ8 and HLA‐DQ8 presented in 29.8% and 10.5% of patients, respectively.^37^ Dehghani et al. found that 72.6% of Iranian children with CD have HLA DQ2/DQ8.^38^ In an Iranian population of first‐degree relatives with CD, the allele frequency of HLA‐DQ2 was 40%, while HLA‐DQ8 was 16%; furthermore, the presence of HLA‐DQ2/DQ8 alleles was 4%. Therefore, the overall frequency of HLA‐DQ2/DQ8 haplotype in Iranian first‐degree relatives with CD was 60%.^39^ In another study in Iran conducted by Fallah et al., the frequency of DQA1*05 and DQB1*02 alleles in CD was reported to be 42.9% and 33.7%, respectively.^40^ A recent study on Iranian children with CD revealed that the frequency of HLA‐DQ8 was 100% among patients.^41^

In the current study, a significant association was also observed between the patient's characteristics including age (>9.5) and gender (female) with the presence of the HLA DQ4/5 genotype. Numerous previous studies have shown the DQ2 role in the mucosal damage intensity^42^, ^43^; such that, it has been reported that in an Iranian study, the 02:01 allele showed a great relationship with Marsh IIIc stage and the function of this allele in the mucosal destruction intensity.^44^ Therefore, further research is suggested to verify the function of these alleles in the CD intensity.

Our study faced a limitation in sample size due to financial constraints. Therefore, future studies should be conducted with a larger sample size and consider additional CD parameters as categorizing factors. These approaches would enable a more accurate interpretation of the correlations between HLA typing findings and various CD parameters.

## 5 CONCLUSION

Our study demonstrates a higher prevalence of HLA‐DQ4 and HLA‐DQ5 in CD patients compared to the control group. Additionally, the elevated relative risk values indicate a significant role of the analyzed alleles in the prevalence of CD among our study participants. It can offer considerable remarks regarding the relationship between HLA‐DQ4/5 genotypes and CD in Southwest Iran, aligning with global reports on the importance of other family members of HLA‐DQ in CD. The present study has opened up a new avenue of research; nonetheless, further investigations are warranted to ascertain the frequency of HLA‐DQ4 and HLA‐DQ5 in diverse populations. This is valuable for enhancing our comprehensive understanding of the putative role of HLA‐DQ4/5 in aiding more definitive decisions in ruling out CD.

## AUTHOR CONTRIBUTIONS

**Ali Keshtkari**: Conceptualization; investigation; supervision; project administration; resources; writing—review and editing; validation. **Marzieh Danaei**: Funding acquisition; writing—original draft; writing—review and editing; supervision; resources; investigation; methodology; conceptualization. **Milad Mollaali**: Writing—original draft; writing—review and editing; validation; visualization; supervision; formal analysis; software; data curation; conceptualization.

## CONFLICT OF INTEREST STATEMENT

The authors declare no conflict of interest.

## ETHICS STATEMENT

This study was performed in line with the principles of the Declaration of Helsinki. Ethical Approval was granted by the Research Ethics Committee of Yasuj University of Medical Sciences (Approval ID: IR.YUMS.REC.1402.113). Informed Consent form was obtained from all the participants (parent or guardian of children).

## TRANSPARENCY STATEMENT

The lead author Milad Mollaali affirms that this manuscript is an honest, accurate, and transparent account of the study being reported; that no important aspects of the study have been omitted; and that any discrepancies from the study as planned (and, if relevant, registered) have been explained.

## Data Availability

The data that support the findings of this study are available from the corresponding author upon reasonable request and with permission from the Research Ethics Committee of Yasuj University of Medical Sciences.
